# Quantification of the binding potential of cell-surface receptors in fresh excised specimens via dual-probe modeling of SERS nanoparticles

**DOI:** 10.1038/srep08582

**Published:** 2015-02-26

**Authors:** Lagnojita Sinha, Yu Wang, Cynthia Yang, Altaz Khan, Jovan G. Brankov, Jonathan T. C. Liu, Kenneth M. Tichauer

**Affiliations:** 1 Biomedical Engineering, Illinois Institute of Technology, Chicago, IL 60616, USA; 2 Electrical Engineering, Illinois Institute of Technology, Chicago, IL 60616, USA; 3 Biomedical Engineering, Stony Brook University (SUNY), Stony Brook NY 11794 USA; 4 Mechanical Engineering, University of Washington, Seattle WA 98195, USA

## Abstract

The complete removal of cancerous tissue is a central aim of surgical oncology, but is difficult to achieve in certain cases, especially when the removal of surrounding normal tissues must be minimized. Therefore, when post-operative pathology identifies residual tumor at the surgical margins, re-excision surgeries are often necessary. An intraoperative approach for tumor-margin assessment, insensitive to nonspecific sources of molecular probe accumulation and contrast, is presented employing kinetic-modeling analysis of dual-probe staining using surface-enhanced Raman scattering nanoparticles (SERS NPs). Human glioma (U251) and epidermoid (A431) tumors were implanted subcutaneously in six athymic mice. Fresh resected tissues were stained with an equimolar mixture of epidermal growth factor receptor (EGFR)-targeted and untargeted SERS NPs. The binding potential (BP; proportional to receptor concentration) of EGFR – a cell-surface receptor associated with cancer – was estimated from kinetic modeling of targeted and untargeted NP concentrations in response to serial rinsing. EGFR BPs in healthy, U251, and A431 tissues were 0.06 ± 0.14, 1.13 ± 0.40, and 2.23 ± 0.86, respectively, which agree with flow-cytometry measurements and published reports. The ability of this approach to quantify the BP of cell-surface biomarkers in fresh tissues opens up an accurate new approach to analyze tumor margins intraoperatively.

Tumor-margin assessment is a critical step in surgical oncology, typically carried out post-surgery using standard histopathology. For breast-conserving surgeries (a.k.a. partial mastectomy or lumpectomy), most institutions define the margin to be “positive” if there are cancer cells on the outer surface of the resected tissue, “close” if there are cancer cells within a defined distance (1–3 mm) from the outer surface, and “negative” if no cells are present within the defined distance from the outer surface of the tumor^1^. There is controversy in this field, as some studies have shown that 1–3 mm margins may not be sufficient for minimizing the risk of tumor recurrence^2^,^3^,^4^, whereas other studies suggest that having no cancer cells at the surface of the excised tissues (“no tumor on ink”) is sufficient^1^,^2^,^5^. Nevertheless, regardless of the margin criteria chosen, it is unequivocal that re-excision surgery is necessary in patients for whom tumor cells are identified at the surgical margin itself (*i.e.*, the surface of the excised tissue). These re-excision procedures are not only time-consuming but also add additional stress and risk for the patient. Approximately 180,000 patients undergo breast-conserving surgery (BCS) each year in the United States and, depending upon the clinic, between 20–60% of these patients require additional surgery owing to incomplete tumor removal as revealed by post-operative pathology^6^,^7^,^8^,^9^,^10^. Considering the high number of re-excision surgeries, there is a need for intraoperative methods of tumor-margin assessment such as wide-field imaging of the entire outer surface of a resected tissue. While wide-field imaging lacks the resolution of microscopic histopathology, it can provide a comprehensive image of the entire surgical margin surface and is therefore unsusceptible to sampling errors introduced by selectively imaging thin sections of the tissue specimen at periodic intervals (bread-loafing), as is done in conventional post-operative histopathology^11^.

Several approaches have been developed for intraoperative surgical guidance, including frozen section histopathology, confocal mosaicing microscopy (CMM)^12^,^13^,^14^, light reflectance spectroscopy (LRS)^15^, auto-florescence lifetime measurement (AFLM)^15^, and touch prep cytology (TP cytology)^16^,^17^. CMM, LRS and AFLM require significant imaging times and are therefore typically performed at spatially distributed points, which can result in sampling errors. In the case of breast cancer, the high fat content of the healthy surrounding tissue makes preparation of frozen sections difficult, and sampling errors are unavoidable when performing frozen-section analyses^18^. Finally, the benefits of TP cytology for guiding breast-tumor resections are still being elucidated^17^. In response, a new intraoperative method is explored here, using surface enhanced Raman spectroscopy nanoparticles (SERS NPs) for wide-area molecular phenotyping.

The detection of cell-surface receptors on cancer cells, with altered expression compared to normal cells^19^,^20^, has long been of interest as a means of discriminating between tumor versus healthy tissue^21^. As a result, numerous cancer-targeted molecular probes have been developed to elucidate the molecular status of cancer. However, a long-standing problem for the molecular imaging of tumors is that the signal obtained from a targeted molecular probe can be affected by many physiological and experimental factors that are completely independent of receptor binding^22^. For example, uneven topical application and rinse removal of unbound probes, uneven detection working distance, variable tissue optical properties, and variable tissue mechanical properties (e.g., diffusion, porosity, interstitial pressure) can all lead to nonspecific and misleading sources of contrast^23^. Therefore, the concentration of a targeted molecular probe is not guaranteed to exhibit a strong correlation with the amount of receptor present, as is the major assumption in most molecular-imaging studies. To account for some of these issues with single-molecular-probe diagnostics, an untargeted probe has been introduced as a means of referencing to control for the aforementioned nonspecific effects for molecular probes that are administered systemically^24^,^25^,^26^,^27^ and topically^23^,^28^,^29^,^30^. Kinetic modeling has previously been evaluated for the analysis of systemically delivered dual probes to estimate “binding potential” (BP)^31^,^32^,^33^,^34^ – a unitless parameter that is directly proportional to receptor concentration^35^. Additional studies with topically applied dual probes have employed a simple ratio of targeted to untargeted probe concentration^23^,^28^,^36^, which has been shown to be faster and simpler, but less accurate and more noise-sensitive than the kinetic-modeling approach^37^,^38^. In the current work, the first application of dual-probe kinetic modeling was carried out for topically applied SERS NP (surface-enhanced Raman scattering nanoparticle) molecular probes.

SERS NPs are silica-shell-encapsulated nanoparticles that can be synthesized to emit spectrally distinct Raman scattering signals upon excitation with a narrowband laser source. SERS NPs have been developed in a variety of “flavors,” each of which generates a unique “fingerprint” spectrum when illuminated with a 785-nm laser source. Since each SERS NP flavor can be identified by its distinct and narrow spectral peaks, the quantity of each NP flavor in a mixture may be determined through a spectral-demultiplexing software algorithm^39^,^40^,^41^,^42^. Here, we demonstrate that these NP molecular probes are ideally suited for so-called “paired-label^27^” or as referred to here, “dual-probe^26^” methodologies to account for nonspecific sources of background contrast. In particular, all of the SERS NP flavors used in the present study can be excited with a single laser and detected within a narrow wavelength band, thereby mitigating errors attributable to differences in tissue optical properties at different wavelengths (as is often the case for fluorophores)^43^. In fact, previous work has demonstrated that untargeted NPs conjugated to isotype-control antibodies are an excellent control for targeted NPs that are conjugated to receptor-specific monoclonal antibodies, with practically identical nonspecific behavior^36^. Utilizing the advantages of dual-probe data analysis, two flavors of SERS NPs (S420 and S440) were used, each possessing its own characteristic Raman fingerprint spectra, where one was targeted to epidermal growth factor receptor (EGFR), a cell-surface receptor overexpressed in many cancers^44^, and the other was left untargeted^36^,^39^. The multiple-time-point data required for kinetic analyses were acquired by a repeated “rinse and detect” procedure (Fig. 1), which required approximately 8–10 minutes per tissue sample.

## Results

Experiments were conducted with tissues from tumor-implant models in mice. Human tumor cell lines that express EGFR at varying levels were xenografted subcutaneously in the flank of nude athymic mice. After approximately 1 month of growth, the tumor implants (ranging in size from 0.5–1.0 cm in diameter) were resected and topically stained for 10 min with an equimolar mixture of EGFR-targeted and untargeted (isotype-control) SERS NPs. The NP concentrations (for both probes) were then measured on the tissues with a spectral-detection device before and after multiple saline-rinse steps to flush out unbound NP concentrations (see methods).

We first present results from a non-kinetic approach for quantifying receptor binding potentials (BP) based on our dual-probe SERS NP data. The estimation of BP calculated with this method is termed BP_Ratio_ = (targeted-untargeted)/untargeted NP concentration, and can be employed on data at a single time point after extensive rinsing. The following section will discuss the use of two different kinetic-modeling approaches to better quantify receptor BPs based on data from multiple rinse steps: (1) a dual-probe model (BP_DPM_) that does not include nonspecific binding and (2) a similar model that DOES include nonspecific binding (BP_DPM-NS_). Finally, we present a few simulations to demonstrate the importance of incorporating nonspecific binding into the kinetic models.

### Ratiometric analyses of SERS-NP binding potential in tissues: BP_Ratio_

Targeted and untargeted SERS NP concentration curves were collected for *n* = 8 samples of U251 tumor implants and *n* = 9 samples of healthy tissue (muscle) and A431 tumor implants on nude athymic mice. Qualitatively, excellent agreement was observed between the concentration curves of the targeted and untargeted NPs in healthy tissue (Fig. 2a), whereas the targeted NP was retained to a greater extent than the untargeted NP in both the U251 (Fig. 2b) and A431 (Fig. 2c) xenografts (proportionately more so in the A431 tissues that most highly express EGFR). In past studies, specifically bound vs. unbound molecular probe concentrations had been quantified through a ratiometric approach: (targeted – untargeted)/untargeted probe signal^23^,^28^,^29^,^30^, which is an estimate of the binding potential (proportional to the concentration of the targeted cell-surface receptor) under equilibrium-like conditions^38^. In the present study, the ratio was typically calculated at the completion of 10 repeated rinses, and the value of this ratio is referred to as the BP_Ratio_. By calculating BP_Ratio_ at all rinse steps (Fig. 2d), statistically significant differences between healthy tissues and both U251 and A431 tumors were observed after the very first rinse (*P* = 0.03 for U251 compared to healthy tissue and *P* = 0.01 for A431 compared to healthy tissue). The difference in BP_Ratio_ between the U251 and A431 tumors was not significant until after the third rinse (*P* = 0.03). After the fourth rinse step, the BP_Ratio_ of the A431 tissues remained relatively stable for all subsequent washes; however, the BP_Ratio_ from the U251 tissues tended to decrease after the fourth rinse. While not statistically significant (*P* = 0.12 by repeated measures ANOVA with “rinse step, subsequent to the fourth rinse,” as a within-subjects variable); this could indicate a preferential rinse removal of the specifically vs. nonspecifically bound targeted NP in this tissue type or could indicate that the binding affinity of the EGFR targeted NPs could vary between the epitopes of EGFR expressed by A431 and U251. The obvious similarity between targeted and untargeted NP concentration-curves in healthy tissue demonstrates the suitability of the chosen isotype antibody-based untargeted SERS NP as an ideal control for the anti-EGFR antibody-based targeted SERS NP, and also corroborates our previous studies demonstrating the superior performance of the untargeted NP as a control for nonspecific effects^36^.

### Kinetic-modeling analyses of SERS-NP binding potential in tissues: BP_DPM_ and BP_DPM-NS_ methods

As described in the Methods, we incorporated two kinetic-modeling approaches to quantify binding potentials (BPs) from experimental measurements of NP concentrations at multiple time points (before and after multiple rinse steps). In particular, we looked at three different tissue types: normal muscle, U251 tumor implants, and A431 tumor implants. A comparison of the different methods of quantifying EGFR expression is provided in Fig. 3, including the targeted SERS NP concentration after the final tissue rinse (Fig. 3a), BP_Ratio_ after the final tissue rinse (Fig. 3b), the dual-probe model estimate of BP (BP_DPM_) (Fig. 3c), and the dual-probe model estimate with nonspecific binding (BP_DPM-NS_) (Fig. 3d). Of the four approaches, only BP_Ratio_ and BP_DPM-NS_ demonstrated statistically significant differences between healthy, U251, and A431 groups (BP_Ratio_: *P*_Healthy-U251_ = 0.0002, *P*_Healthy-A431_ = 4 × 10^−6^, *P*_A431-U251_ = 2 × 10^−6^; and BP_DPM-NS_ : *P*_Healthy-U251_ = 0.0002 *P*_Healthy-A431_ = 0.0002 *P*_A431-U251_ = 0.0160) with mean BP_Ratio_ of 0.00 ± 0.06, 0.33 ± .18, and 1.03 ± 0.11 for healthy, U251, and A431 tissue, respectively; and mean BP_DPM-NS_ of 0.06 ± 0.14, 1.13 ± 0.40, and 2.23 ± 0.86. The failure of BP_DPM_ in indicating different EGFR concentrations of the different tissue types (Fig. 3b) further confirms that nonspecific binding is a major factor for these SERS NP-based molecular probes.

### Simulations to demonstrate the importance of modeling nonspecific (NS) binding

Increasing levels of nonspecific binding were added in simulated kinetic datasets of targeted and untargeted SERS NPs in tissues. These simulations demonstrated that both BP_Ratio_ and BP_DPM_ were far more sensitive to variations in nonspecific binding than BP_DPM-NS_ (Fig. 4a), with higher levels of nonspecific binding yielding larger errors in estimated BP in all cases (Fig. 4b). When applying BP_Ratio_ to simulated data with experimental levels of nonspecific binding (*k*_5_ = 0.16 min^−1^ and *k*_6_ = 0.06 min^−1^) and BP values expected in healthy, U251 and A431 tissue (0, 1 and 3, respectively), significant underestimations in BP were observed (Fig. 4c). In contrast, BP_DPM-NS_ was capable of accurately predicting simulated BP levels despite substantial levels of nonspecific binding (Fig. 4d).

### Validation of results

The accuracy of our results was evaluated by a flow-cytometry analysis of the tumor cell lines to estimate the relative amounts of EGFR expression. The results suggest, on average, that the A431 cell line expresses 3.89 times more EGFR than the U251 cell line (on a per cell basis). Taking into account the fact that in subcutaneous nude mouse xenografts, U251 cells grow in denser populations by a factor of 30%^34^, A431 is expected to express 3.05 ± 0.88 times more EGFR by volume than U251. In comparison, the BP_Ratio_ measures of EGFR expression estimated 3.12 ± 0.07 times more EGFR density in A431 compared to U251, matching well with the flow-cytometry estimate, while the BP_DPM-NS_ measures of EGFR expression estimated 1.97 ± 0.32 times more, significantly underestimating the flow cytometry estimate (*P* = 0.02). However, it should be noted that flow-cytometry estimates of EGFR expression of the A431 tumor line have been shown to substantially overestimate EGFR expression compared to quantitative immunofluorescence staining of A431 tumor xenografts^45^. In fact, when comparing A431 to U251 EGFR expression through quantitative immunofluorescence staining, the A431 line was estimated to have only 2.1 ± 0.3 times more EGFR^34^,^45^, much closer to the value determined by BP_DPM-NS_ compared to BP_Ratio_ in the present study. The variability in “gold-standard” estimates of EGFR expression could be a result of the inability of flow-cytometry to predict the number of EGFR receptors “available” for binding in tissue. Flow-cytometry indicates total number of EGFR per cell; whereas *in vivo*, the binding of NPs to EGFR proteins could be impeded by adjacent cells or extracellular matrix not included in the flow-cytometry assay^35^. Additionally, the discrepancy could owe to a greater binding-site barrier in the higher EGFR-expressing A431 tumors^46^. Despite these possible factors, the relatively good agreement between the flow cytometry/literature results, and both BP_Ratio_ and BP_DPM-NS_, suggests that either measure could be used to provide an estimate of relative EGFR concentrations.

## Discussion

In the present study, we compared various approaches for quantifying the binding potential (BP: proportional to receptor densities) of cell-surface receptors in fresh tissues based on the retention kinetics of dual NP probes (targeted and untargeted) that were simultaneously applied as an equimolar mixture on excised tissues and then rinsed away in multiple stages. The most conventional molecular-detection approach of simply measuring the signal of a targeted molecular probe (in this case a SERS NP) after repeated rinses showed no considerable differences in retention between all groups of tissues, whether tumor or normal (Fig. 3a). This finding is in part attributable to a significant amount of unbound and/or nonspecifically bound NPs that are retained in all tissue types and suggests that even with substantial and repeated rinsing of the tissue, the occurrence of delivery variability, unbound NP concentrations and/or nonspecifically bound NPs can overshadow the signal from NPs that are specifically bound to their targets, at least with the type of large SERS NP/antibody-based molecular probes used in the present study. It is possible that smaller molecular probes could be washed from the tissue more readily, thereby allowing a single targeted probe alone to be more indicative of receptor concentration. However, even if it is possible to completely wash away all probe that is not specifically bound, the initial delivery of the probe into the tissue could still be highly variable and often depends on the physical and morphological characteristics (e.g. permeability) of the tissue specimens being evaluated. In addition, the signal measured from the tissue could be heavily dependent on the optical properties of the tissue as well as experimental conditions such as working distance and angle of the detection device, thereby making it difficult to distinguish true molecular contrast from nonspecific (nonchemical) sources of contrast^47^. The dual-probe SERS NP approach described in the current study is not subject to any of these limitations. By referencing the targeted NP concentration to a co-administered untargeted NP concentration, variability in targeted NP delivery efficacy, nonspecific binding, and other “common-mode” sources of noise and background contrast described above can be accounted for^34^. Furthermore, by utilizing SERS NPs with distinct spectrally coded signals that can be excited at a single illumination wavelength, it is possible to 1) avoid differences in tissue optical properties between the two NPs, and 2) add additional NPs targeted to other cancer-specific receptors to develop a more comprehensive molecular analysis of the tissue and therefore a better discrimination of cancer vs. healthy tissue^36^. This final point could be particularly important for cancers presenting heterogeneous molecular phenotypes^48^, where imaging of a single receptor may not highlight the entire tumor.

Four methods of estimating EGFR concentrations in normal muscle (no EGFR), U251 tumor (moderate EGFR) and A431 tumor (high EGFR) tissues were compared: 1) targeted NP concentration after 10 rinses, BP estimation via a ratio of targeted to untargeted NP concentration (BP_Ratio_) after 10 rinses, BP estimation via dual-probe model analysis without nonspecific binding (BP_DPM_), and BP estimation via dual-probe model analysis WITH nonspecific binding (BP_DPM-NS_). Only BP_Ratio_ and BP_DPM-NS_ estimates of EGFR receptor concentration were capable of discriminating between different tissue types with known differences in EGFR expression (Figs. 3b,d).

With BP_Ratio_ being much simpler to apply and requiring little computational effort, it would seem like the more attractive approach; however, it should be noted that BP_DPM-NS_ values were about twice as large as BP_Ratio_ values. Simulations suggest that this is an expected finding in the presence of nonspecific binding, where BP_Ratio_ is expected to significantly underestimate the true value (Fig. 4c). If the level of nonspecific binding is similar in all tissues being compared, then this error will be cancelled out if only relative differences in EGFR expression are of interest. In the present study, the similarity in the ratio of A431:U251 EGFR expression measured by BP_Ratio_ and BP_DPM-NS_ suggests that the level of nonspecific binding was indeed similar between A431 and U251 tissues; however, this would need to be tested on a case-by-case basis and it would be far more reliable to measure BP_DPM-NS_, which is not sensitive to differences in the extent of nonspecific binding.

Another obvious difference between BP_Ratio_ and BP_DPM-NS_ was that BP_Ratio_ exhibited smaller variance for the A431 tissue group. This would also be preferable. However, simulations predicted that BP_DPM-NS_ would have better precision than the BP_Ratio_ [± 4% s.d. of the mean compared to ± 12% s.d. of the mean, respectively, at moderate levels of receptor expression (Fig. 4c,d)]. The observed variability in tissue samples (± 30% compared to ± 50% for BP_Ratio_ and BP_DPM-NS_, respectively) demonstrates that experiment variability is at least 3 times larger than variability in parameter estimation. Thus, it is possible that there is true EGFR expression variability within and between the various tissue samples used in the present study^34^. It is also possible that the greater variance in BP_DPM-NS_ could be associated with minor inconsistencies in the rinse steps between measurements, or perhaps unmodeled NP diffusion^49^,^50^. Further studies will be required to fully elucidate the source of the increased variance observed with BP_DPM-NS_ and to determine if spatial diffusion would need to be modeled^50^. However, it should be stressed that the inclusion of more fitting parameters (diffusion) could result in increased instability in parameter estimation and fit errors will be affecting, thus altering the current error analysis.

The novel kinetic-modeling approach (BP_DPM-NS_) developed in the present study has the potential to have a substantial impact on the medical community. For example, the dual-probe SERS NP analysis technique could help improve patient outcomes and/or reduce the rate of re-excision surgery by allowing surgeons to better ascertain whether the margins of excised tissues are free of residual tumor (clean margins). Further, with future developments allowing the determination of BPs of multiple cancer-specific receptors – using a cocktail of many targeted SERS NPs and one untargeted NP to account for nonspecific effects – this approach will offer increased accuracy for tumor detection in spite of the large degree of molecular heterogeneity amongst tumors. The added complexity of a kinetic-modeling approach, compared to the simpler and faster ratiometric quantification approach, may be justified in a clinical setting if it increases the sensitivity and specificity of tumor detection at the surgical margins. Future clinical studies will be necessary to investigate these trade offs.

## Methods

### Animal experiments

All animal experiments were in accordance with guidelines approved by the Institutional Animal Care and Use Committee (IACUC) at Stony Brook University. Six nude male mice at 7–9 weeks-of-age (*n* = 6) were inoculated subcutaneously with 3 × 10^6^ U251 human glioma cells and 1 × 10^6^ A431 human epidermoid cells (American Type Culture Collection (ATCC), Manassas, VA), on the right and left flanks, respectively. Once both tumors were greater than 5 mm in size, the mice were euthanized and the tumors and an equivalent piece of muscle tissue (control “healthy” tissue from the thigh) were resected.

### Tissue specimen analysis

For *ex vivo* analyses, the tissue specimens were cut into small pieces of about 5–10 mm followed by hydration in Fluorescence Activated Cell Sorting (FACS) buffer for the acquisition of a spectral background of the unstained tissue, and subsequent NP staining. The NP staining solution consisted of an equimolar concentration of EGFR-targeted as well as isotype-control NPs (both 150 pM) along with 1% BSA to reduce nonspecific binding. Tissue pieces were stained with 15 μL of the staining solution for 10 min at which point Raman signal was measured with a Raman-detection device^36^. The specimens were then sprayed with 0.1 mL of Phosphate Buffered Saline (PBS) solution and dabbed dry. This step removes a significant portion of unbound NPs from the specimens. The specimens were then imaged again and this process of rinsing and imaging was repeated 10 times. The PBS solution is isotonic and is also non-toxic to cells, so it is used for rinsing purposes. For calibration purposes, a 4-μL droplet of the original equimolar SERS NP mixture was placed on a tissue sample and a spectrum was acquired with the same detection parameters (working distance and angle). The concentrations of the targeted and untargeted NP flavors were both set as 150 pM to calibrate the data sets, thereby enabling conversion from spectral measurements (NP weights) to NP concentrations. The signals from multiple types of SERS NPs were demultiplexed to determine their respective concentrations, using approaches described in detail previously^36^,^39^.

### Kinetic analyses

Analyses were performed on the multiple-rinse-step data using four competing approaches to estimate relative EGFR concentration in the healthy, U251, and A431 tissues via the “binding potential” (BP) kinetic modeling parameter, which is equivalent to affinity of a targeted probe for its targeted receptor and the concentration of targeted receptor^35^. BP can be conceptualized at the ratio of specifically bound probe to unbound probe at equilibrium in a tissue (i.e., a BP = 1 infers that the concentration of probe in the region is divided evenly between bound and unbound states). Since BP is proportional to probe affinity, a general scale defining what is a high or a low BP cannot be produced; however, to provide some scale, a probe with an affinity of 1 nM^−1^, will be approximately 3 for high EGFR expressing tumor lines and approximately 1 for moderate EGFR expressing tumor lines^34^. The methods of estimating BP or EGFR concentration evaluated in the present study were: 1) Targeted NP concentration at the end of the rinsing cycle (conventional single-probe molecular imaging); 2) BP estimation based on a ratio of targeted to untargeted NP concentrations (BP_Ratio_, Supplementary Eq. 7); 3) BP estimation using a dual-probe model (BP_DPM_) (Supplementary Eqs. 1 & 2); and 4) BP estimation using a dual-probe model with nonspecific binding (BP_DPM-NS_) (Supplementary Eqs. 3 & 4). Approaches 1 and 2 are straightforward; for the second two approaches, non-negative least-squares fitting was performed in a two-step process using in-house software developed in MATLAB 2011a (Mathworks, Natick, MA). For BP_DPM_, the untargeted NP concentration curve was fit with Eq. 2 to estimate the rinse rate parameter, *F*. This parameter was then incorporated into Eq. 1 when fitting the concentration curve of the targeted NP to estimate *k*_3_/*k*_4_ = BP. A similar process was carried out for BP_DPM-NS_. Specifically, the untargeted NP concentration curve was fit with Supplementary Eq. 3 to estimate *F*, *k*_5_, and *k*_6_. These three parameters were then incorporated into Supplementary Eq. 4 for fitting the targeted NP concentration curve to estimate *k*_3_/*k*_4_ = BP (see Supplementary Information) to estimate a relative measure of EGFR concentration. When fitting data with Supplementary Eqs. (4), regularization was applied, such that noise amplification did not occur, using a randomized column Kaczmarz method with Tikhonov regularization for the computation of the arbitrary coefficients^51^.

### SERS nanoparticle signal measurements

The SERS NPs used in the present study were purchased from Cabot Security Materials Inc. SERS NPs were conjugated with fluorophores (Cyto 647-maleimide, Cytodiagnostics Inc, NF647-3-01) for flow cytometry as well as an anti-EGFR monoclonal antibody (mAb, Thermo Scientific, MS-378-PABX) or an isotype control mAb (Thermo Scientific, MA110407). A description of the SERS NPs and the detailed conjugation protocol employed in the present study can be found in a previous publication^36^.

**Figure 1:**
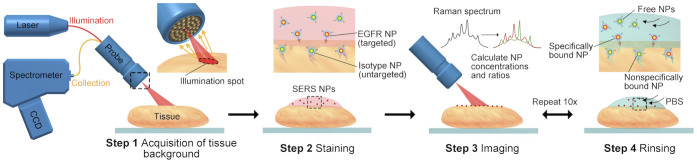
Stepwise demonstration of a tissue-rinsing and spectral-detection procedure performed using SERS NPs. After measuring a tissue background spectrum, the resected tissue was initially stained with an equimolar mixture of targeted and untargeted SERS NPs (Step 2). Spectroscopy of the tissue was then carried out at regular intervals (~30 s) between repeated rinse steps with phosphate buffered saline (PBS) (steps 3 & 4).

**Figure 2:**
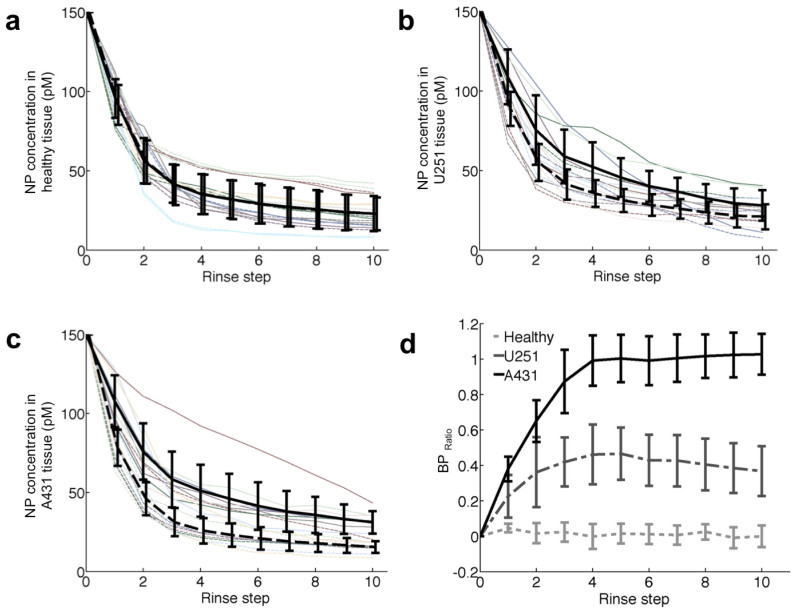
Targeted and untargeted surface-enhance Raman scattering nanoparticle (SERS NP) retention dynamics. The NP concentration curves obtained from (a) healthy normal tissue, (b) U251 xenografts and (c) A431 xenografts, are presented as a function of the number of tissue rinses. The thin solid lines represent the concentration of targeted NPs; the thin dashed lines indicate untargeted NPs; the black bold solid lines represent the mean ± sd concentration of targeted NPs (*n* = 8 tissue samples for U251, *n* = 9 for the healthy and A431), and the dashed bold curves represent the mean ± sd concentration of the untargeted NPs. (d) Change in the ratio-estimated binding potential (BP_Ratio_), where BP_Ratio_ = (targeted-untargeted)/untargeted NP concentration, as a function of multiple rinse steps.

**Figure 3:**
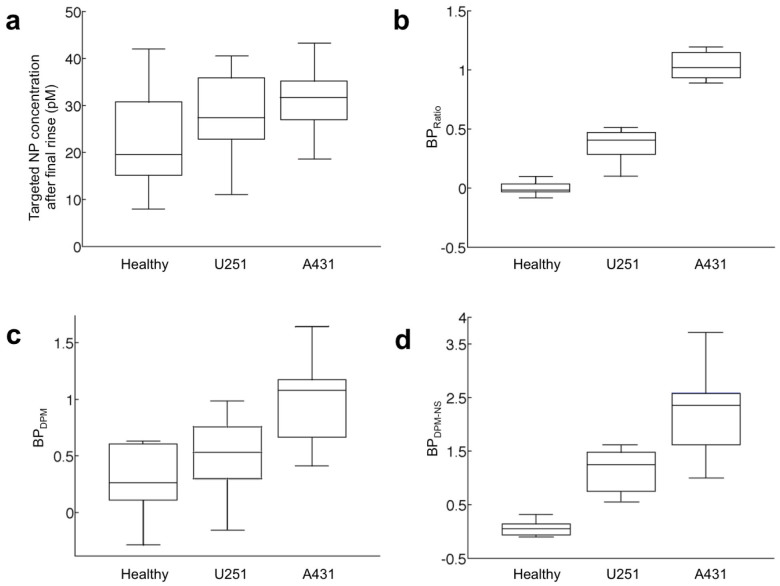
Comparison of approaches for estimating EGFR binding potential (BP) in three different tissue types: healthy (no EGFR), U251 (moderate EGFR) and A431 (high EGFR). Note that BP is proportional to receptor density (a) A boxplot of the concentration of EGFR-targeted SERS NPs after the final tissue rinse (after 10 rinses). (b) A boxplot of BP estimates using the ratio approach [BP_Ratio_ = (targeted-untargeted)/untargeted NP concentration] after 10 tissue rinses. (c) A boxplot of BP estimates using the dual-probe model (BP_DPM_), which does not incorporate nonspecific binding. (d) A boxplot of BP estimates using the dual-probe model with a nonspecific binding compartment (BP_DPM-NS_). The boundaries of the boxes represent the upper and lower quartiles (25% of data greater or less than these boundaries), the horizontal lines represent the medians and the whiskers represent the maximum and minimum data points excluding outliers (more or less than 1.5 times the upper or lower quartile, respectively).

**Figure 4:**
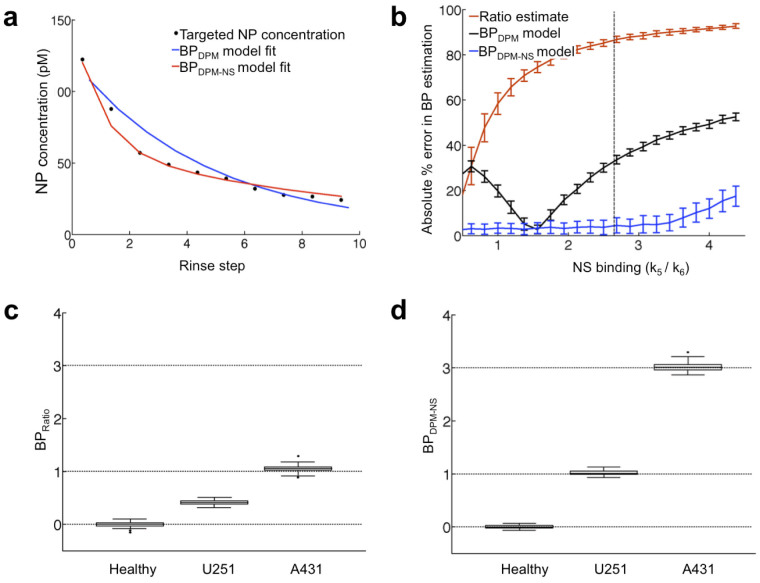
Importance of incorporating a nonspecific-binding compartment into a kinetic model for estimating binding potential (BP, proportional to receptor concentration). (a) Demonstration of a representative concentration curve for a targeted SERS NP in a U251 tissue sample (black dots). Dual-probe model BP (BP_DPM_; blue line) and dual-probe model with nonspecific binding BP (BP_DPM-NS_; red line) fits of the data are presented. (b) An error-analysis plot from simulation studies demonstrating increased absolute error in BP estimation with increased nonspecific binding (NS binding: quantified by ratio of NS association and dissociation rate constants, *k*_5_/*k*_6_) for all three estimates of BP: BP_Ratio_ after final rinse (red data), BP_DPM_ (black data) and BP_DPM-NS_ (blue data). The dotted line represents the average NS binding ratio obtained from the 26 tissue samples. (c) and (d) Boxplots of BP_Ratio_ and BP_DPM-NS_ estimates from simulated data for levels of BP comparable to that of the healthy, U251 and A431 tissues used in the present study (0, 1, and 3, respectively, represented by horizontal dashed lines) and an NS binding ratio (*k*_5_/*k*_6_) = 2.67. The boundaries of the boxes represent the upper and lower quartiles (25% of data greater or less than these boundaries), the horizontal lines represent the medians and the whiskers represent the maximum and minimum data points excluding outliers (more or less than 1.5 times the upper or lower quartile, respectively).

A miniature Raman-detection device has been developed to quantify the concentration and concentration ratios of SERS NPs applied on tissues^52^. A 10-mW 785-nm diode laser was used to illuminate the tissue with a spot size of ~500 μm. A device holder was used to maintain a constant working distance (5 mm) and device angle (45°) in order to minimize specular reflections (Fig. 1). A custom spectrometer (Bayspec Inc.) was used to disperse the collected signal onto a cooled deep-depletion spectroscopic CCD (Andor Newton, DU920P-BR-DD). A detector integration time of 0.5 s was used. A conventional direct classical least squares (DCLS) demultiplexing method was employed to calculate the weight of targeted and untargeted NPs from a raw spectrum^39^,^40^,^41^, since the Raman spectra of both NPs were sufficiently distinct (Supplementary Information). The concentrations and concentration ratio were calculated based on a calibration measurement with a stock mixture of targeted and untargeted NPs applied on tissue (refer to **Tissue specimen analysis**). Linearity and accuracy of the correlation between SERS NP demultiplexing estimation of NP concentration and known concentrations was tested out in solutions with 1 to 400 pM of equimolar EGFR-targeted and untargeted NPs (Supplementary Information). The maximum concentration used in the tissue imaging was 150 pM.

### Simulation experiments

To test out the models as described in the **Theory** section (Supplementary), NP concentration curves were simulated for both the targeted and untargeted SERS NPs using equations 3 and 4 in Supplementary, respectively. In the simulations, the BP was kept constant at 2.17 (average BP for A431) while a range of nonspecific binding levels was simulated by varying the “association” rate constant of nonspecific binding, *k*_5_, while keeping the “dissociation” rate constant, *k*_6_, at 0.06 min^−1^ (average from data fitting). The error bars were obtained from 100 iterations with 0.48% noise added for each simulation (noise estimated from experimental data). *k*_4_ was taken to be consistent with the dissociation rate of EGFR binding^37^. In the simulations it was assumed that the targeted and untargeted NPs were ideal and therefore the rate constants *k*_5_ and *k*_6_ are equivalent for both of them (note that the similarity between the targeted and untargeted NP concentration curves in healthy tissue supports this assumption in the current study). Since this is a novel approach, no physiological data exists; however, according to *ex vivo* flow-cytometry results, the bound receptor concentration for A431 was found to be 3.05 times that of U251. Moreover, it is also known that tumors have a much higher EGFR concentration than the healthy muscle tissue^20^. As discussed earlier, the BP is proportional to the EGFR concentration. The BP was taken to be 0.0005, 1 and 3 for tissue with low, moderate and high EGFR concentration, respectively, corresponding to healthy, U251 and A431 tissue. All the simulated NP concentration curves were generated corresponding to 10 rinses according to the procedure described in the **Tissue specimen analysis** section.

For the best representation of the actual tissue data, a noise-detection technique was applied to approximate the percentage of noise in the uptake curves. At each rinse step the targeted and untargeted NP concentrations were extracted and were fitted to a clean signal to determine the level of noise that was given by the following equation: 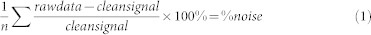
where the clean signal was obtained by fitting the data from all the tissue pieces with a smooth curve obtained by the BP_DPM-NS_ model for the targeted NP concentration curve and the untargeted NP concentration curve. Mean noise was 0.48% in the experimental data. Thus, Gaussian noise of 0.48% was added to all the simulated curves before application of the BP estimation approaches to the simulated NP concentration curves. 100 simulation iterations were performed, and BP_Ratio_, BP_DPM_ and BP_DPM-NS_ were compared for accuracy and precision.

### Statistics

All statistics in the present study were carried out with the statistical package SPSS (IBM, Armonk, NY, USA). A one-way ANOVA was used, with “tumor line” as a between-subjects factor (*n* = 9 for healthy, *n* = 8 for U251 cell line and *n* = 9 for A431 cell line), to compare BPs in each group with alpha = 0.05. Homogeneity of variances was assessed by Levene Statistics. If the Levene statistic led to *P* < 0.05, demonstrating non-homogeneous variance between groups, a Tamhane-corrected post-hoc analysis was performed. If *P* > 0.05, we used Bonferroni post-hoc analysis. The Shapiro-Wilk test was used to determine that all distributions were normal.

## Author Contributions

L.S. performed kinetic modeling, analyzed the data, and wrote the manuscript. Y.W. designed and performed the imaging experiments, and wrote the manuscript. A.K. assisted with the imaging experiments. C.Y. helped to develop the kinetic models. J.G.B. helped to supervise the project. K.M.T. supervised the kinetic modeling, initiated the project, analyzed data, and wrote the manuscript. J.T.C.L. initiated the project, supervised and designed the imaging experiments, analyzed the data, and wrote the manuscript.

## Supplementary Material

Supplementary Information Supplementary Information
