# Supplementary material for: Quantification of the binding potential of cell-surface receptors in fresh excised specimens via dual-probe modeling of SERS nanoparticles
Source: Sci Rep. 2015 Feb 26;5:8582. doi: 10.1038/srep08582 (PMC4341215; doi:10.1038/srep08582)
Supplement: Supplementary Information [file srep08582-s1.pdf]

Supplemental Information: Quantification of the binding potential of cell-surface receptors in fresh excised specimens via dual-probe modeling of SERS nanoparticles

Lagnojita Sinha,[1,2,†] Yu Wang,[3,4,†] Cynthia Yang,[1] Altaz Khan,[3,4] Jovan G. Brankov,[2] Jonathan T. C. Liu,[3,4] Kenneth M. Tichauer[1,*]

[1]*Biomedical Engineering, Illinois Institute of Technology, Chicago, IL 60616, USA*

[2]*Electrical Engineering, Illinois Institute of Technology, Chicago, IL 60616, USA*

[3]*Biomedical Engineering, Stony Brook University (SUNY), Stony Brook NY 11794 USA.*

[4]*Mechanical Engineering, University of Washington, Seattle WA 98195, USA.*

[†]Authors contributed equally to the publication

Surface enhanced Raman scattering nanoparticle (SERS NP) signal linearity

To ensure and demonstrate linearity between demultiplexed SERS NP signals and the respective concentrations of the targeted and untargeted SERS NP, NP dilutions were carried out for known equimolar concentrations of EGFR-targeted and untargeted NPs from 1 to 400 pM. Multispectral Raman data were collected using the system described in the Methods at each concentration. The data were demultiplexed (**Suppl. Fig. 1a**) and converted to concentrations of both targeted and untargeted NPs (**Suppl. Fig. 1b**). Both the measured concentrations and the concentration ratio show good linearity in the range of 1 to 400 pM (**Suppl. Fig. 1c**).

Specificity of anti-epidermal growth factor receptor (EGFR) targeted SERS NPs

The specificity of targeted SERS NPs to EGFR was evaluated in 6 U251 and 6 A431 excised tissue samples. Subcutaneous tumor xenografts were surgically removed from mice and cut in half. One half of each tumor was pre-incubated with 100-μL of an EGFR antibody (~10 mM in PBS) for 3 hours to block the EGFR receptors on the tissue surface. The blocked tumor section and the matching section were then

topically stained with an equimolar mixture of EGFR-targeted and isotype NPs (each 150 pM) for 10 min, rinsed with PBS, and imaged immediately. The concentration ratio of EGFR-targeted NPs vs isotype NPs was estimated from SERS demultiplexing for all four tumor-groups: U251 blocked, U251, A431 blocked, and A431 (Suppl. Fig. 2). The blocked A431 and U251 tumors show an evident reduction in the concentration ratio of the targeted vs. nontargeted NPs, indicating that the EGFR NPs are specific to the EGFR receptors ($P < 0.001$).

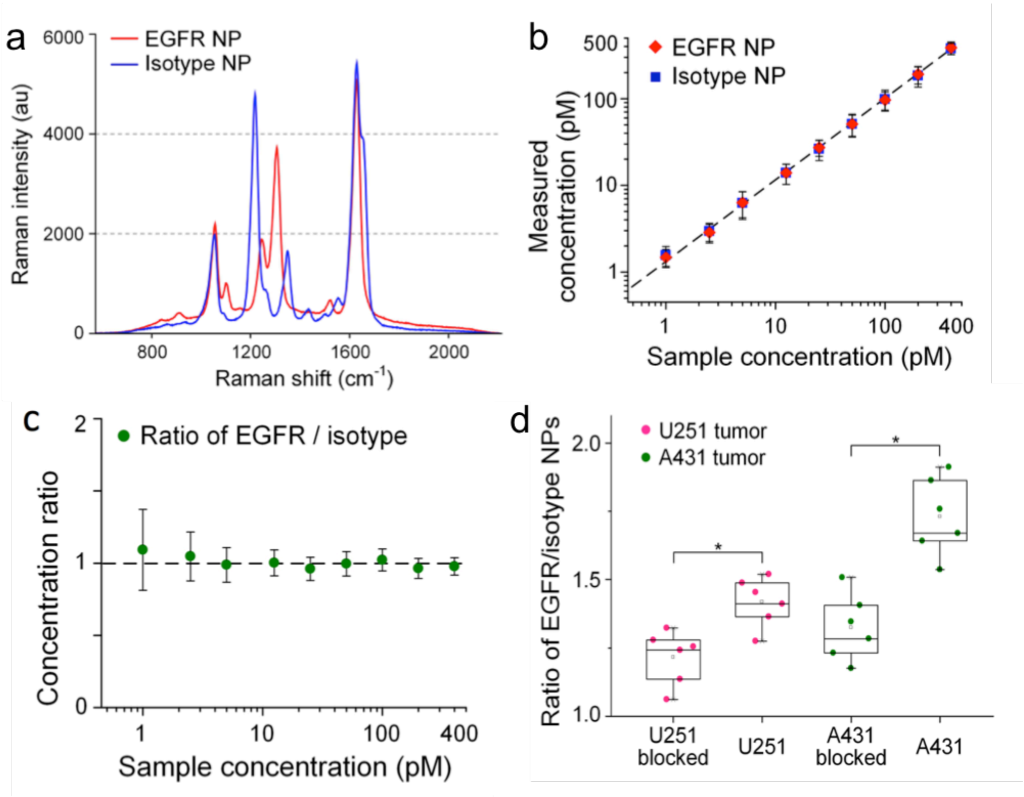

Supplementary figure 1 Reproducibility and linearity of spectral measurements. (a) SERS spectra of the EGFR-targeted NPs and the isotype-control NPs used in this study. (b) Measured concentration and (c) concentration ratios of EGFR-targeted NPs and isotype NPs [mixed in an equimolar ratio (1:1)] plotted against known sample concentration from 1 to 400 pM. Error bars represent the standard deviation. (d) Ratios of estimated EGFR targeted to untargeted SERS NP concentration measured in U251 and A431 excised tumor specimens with and without EGFR blocking by 10 mM unlabeled anti-EGFR antibody pre-incubation. *$P < 0.001$.

Kinetic modeling

The kinetic analysis of dual-probe SERS NPs that are topically applied on tissue sections requires determination of an optimal simplified compartment model to approximate the complex physiological environment without compromising key factors that could influence the NPs' kinetics. Thus, the dominant states the NP may be in must be considered while avoiding complexities like rare molecular interactions that would only complicate the model while scarcely influencing the kinetics. As mentioned, the studies carried out in this research focus on the topical delivery of targeted and untargeted SERS NPs on surgically resected tissues; therefore, the aim was to develop a robust kinetic model incorporating the dual-probe methodology that would accurately quantify the binding potential, which is proportional to the targeted receptor concentration of the tissue biopsy shavings[1] to effectively identify tumor vs. healthy tissue based on cancer-specific receptor expression[2]. The models developed are discussed below.

Dual-probe model estimate of binding potential (BP_{DPM})

In the dual-probe model used to estimate binding potential (BP, proportion to molecular probe target concentration) the main compartments considered are the:

- *Free compartments*: both the targeted and untargeted NPs may be free (unbound) in the interstitial space. The concentration of NP in free space is represented by "C_f" for targeted NP and "C_f'" for untargeted NP
- *Specifically bound compartment*: The targeted NPs may become bound to the receptors on the cell surface. "C_b'" represents the concentration of the NPs that are bound to the receptors.

Based on these stipulations, a one-compartmental model is employed to characterize the untargeted NP tissue concentration curves in response to repeated rinsing (**Supplementary Fig. 1a**) and a two-compartmental model is employed to characterize targeted NP tissue concentration curves

(**Supplementary Fig. 1b**). When these one- and two- compartmental models are solved as shown in the **Detailed Computation** section, the following solution is obtained:

$$\begin{bmatrix} C_f(t) \\ C_b(t) \end{bmatrix} = c_1'\left[\mathbf{v}_1'\right]e^{\lambda_1' t} + c_2'\left[\mathbf{v}_2'\right]e^{\lambda_2' t},$$

$$\text{with } \begin{bmatrix} c_1' & c_2' \end{bmatrix} = \begin{bmatrix} \mathbf{v}_1' & \mathbf{v}_2' \end{bmatrix}^{-1} \cdot \begin{bmatrix} C_0 \\ 0 \end{bmatrix}. \quad (1)$$

$$\text{and } C_{t1}(t) = C_0 e^{-Ft}, \quad (2)$$

where C_0 represents the initial concentration of NP, which is assumed to be same for both the targeted and untargeted NPs, and F is a constant associated with the flush rate of the topical stained tissues. The variables λ_1'' and λ_2'' are the eigenvalues, $\mathbf{v}_1''$ and $\mathbf{v}_2''$ are the eigenvectors, and c_1'' and c_2'' are the constants of the simultaneous differential equations in Supplementary Fig. 1b. A detailed definition of these values is shown in the **Detailed Computation** section and are mathematical expressions based on C_0, F, and k_3 and k_4, the latter of which are rate constants governing receptor binding and dissociation.

Initial estimates of BP using the dual-probe model defined in Eqs. (1) and (2) (BP_{DPM}) suggested that a third, "nonspecific binding/retention" state of the targeted NP was likely. Both flow-cytometry and tissue-staining experiments have also indicated that our topically applied untargeted SERS NPs exhibit significant nonspecific binding and/or retention that should be modeled as a separate compartment, as described in the next section.

Dual-probe model with nonspecific binding compartment estimate of binding potential ($BP_{DPM\text{-}NS}$)

In response, a new model was developed to account for nonspecific binding by adding a second compartment to the untargeted NP model (**Supplementary Fig. 2a**) and a third compartment to the targeted NP model (**Supplementary Fig. 2b**). The concentrations of NPs nonspecifically retained are called C_{ns} for the targeted NP and $C_{ns'}$ for the untargeted NP. k_5 and k_6 are likewise the first-order rate

constants of association and dissociation of the targeted and untargeted NPs associated with nonspecific binding/retention.

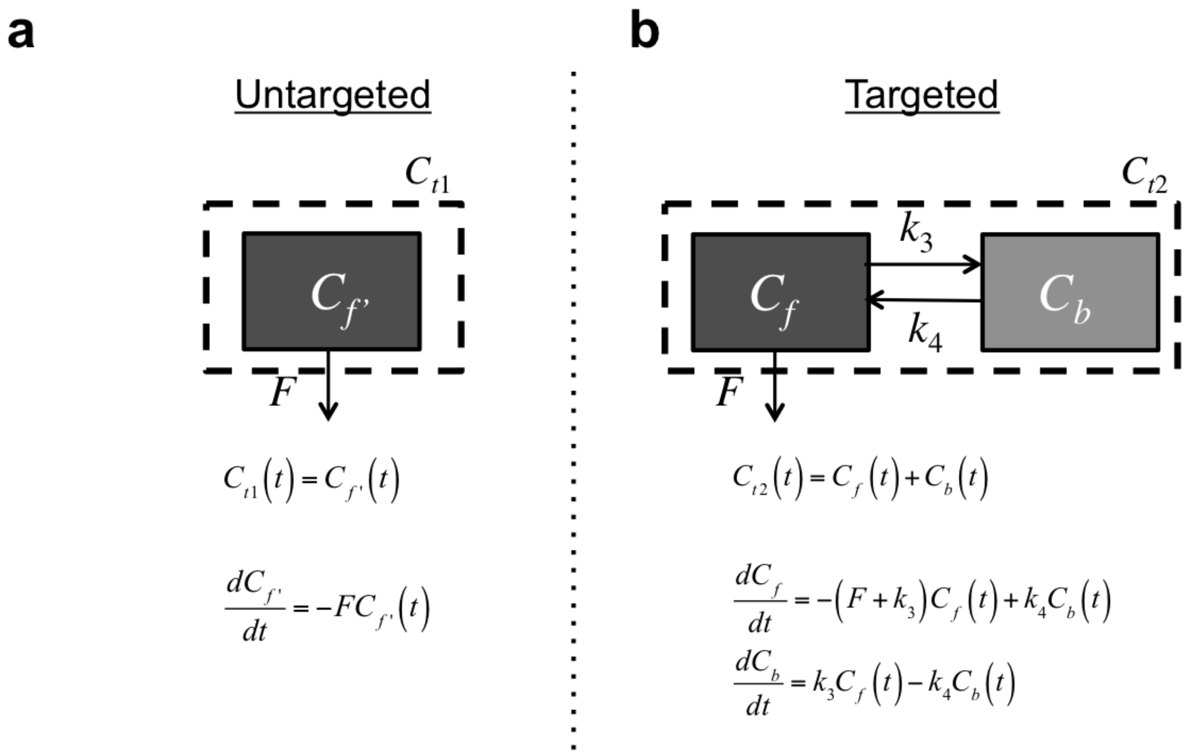

Supplementary figure 2 Dual-probe models for *ex vivo* classification of tissue using topically applied surface enhanced Raman scattering nanoparticles (SERS NPs). (**a**) One-compartment model to approximate untargeted NP concentration curves in response to repeated rinsing. (**b**) Two-compartment model to approximate targeted NP concentration curves in response to repeated rinsing. The concentration of molecules in free space is represented by C_f for targeted NP and $C_{f'}$ for untargeted NP. C_b represents the concentration of the NPs that are bound to the receptors. Variables k_3, k_4, and F are the rate-constant parameters representing NP flux transfer between different compartments.

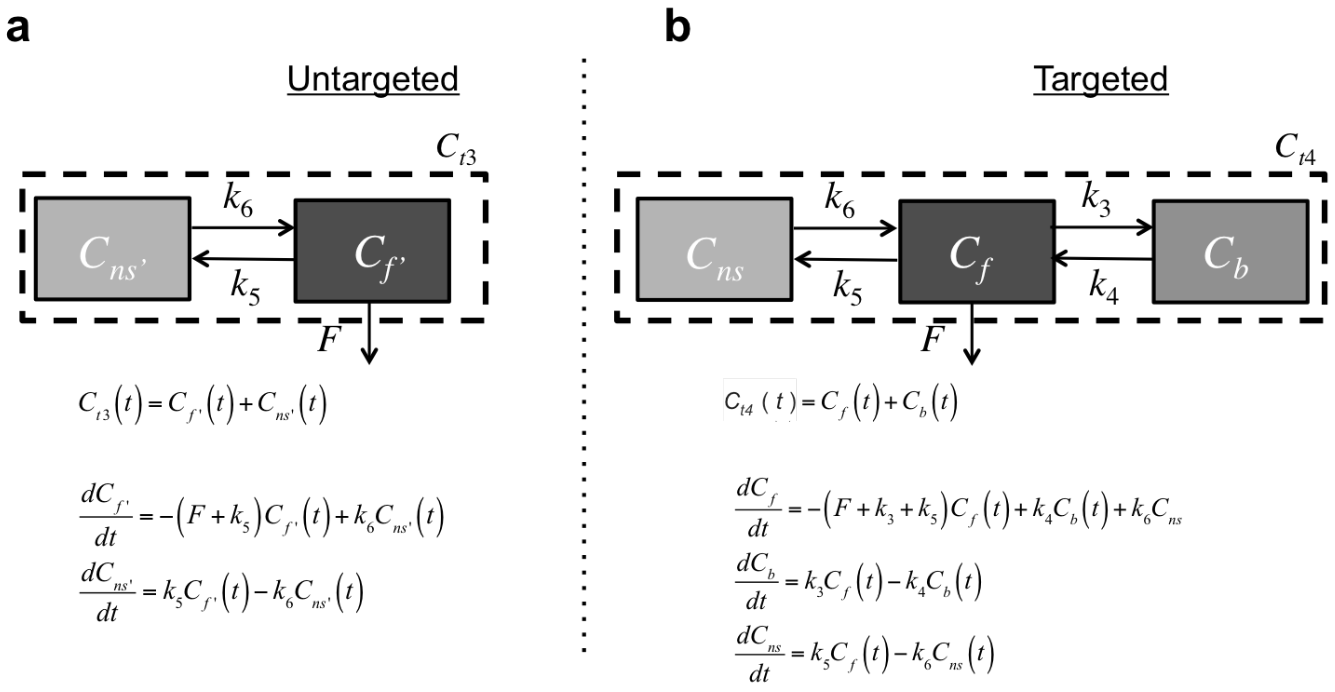

Supplementary figure 3 Dual-probe models with nonspecific binding for *ex vivo* classification of tissue using topically applied SERS NPs. (**a**) Two-compartment model to approximate untargeted NP concentration curves in response to repeated rinsing. (**b**) Three-compartment model to approximate targeted NP concentration curves in response to repeated rinsing. The concentration of molecules in free space is represented by C_f for the targeted NP and $C_{f'}$ for the untargeted NP. C_b represents the concentration of the NPs that are bound to the receptors. The concentrations of NPs that are nonspecifically bound are denoted as C_{ns} for targeted NP and $C_{ns'}$ for the untargeted NP. k_3, k_4, k_5, k_6, and F are the rate constants for the NP flux transfer between different compartments.

The solution for the untargeted NP compartmental model is shown in the **Detailed computation** section, and the result for the untargeted NP two-compartment model is given below[3]:

$$\begin{bmatrix} C_{f'}(t) \\ C_{ns'}(t) \end{bmatrix} = c_1'[\mathbf{v}_1']e^{\lambda_1' t} + c_2'[\mathbf{v}_2']e^{\lambda_2' t},$$
$$\text{with} \begin{bmatrix} c_1' & c_2' \end{bmatrix} = \begin{bmatrix} \mathbf{v}_1' & \mathbf{v}_2' \end{bmatrix}^{-1} \cdot \begin{bmatrix} C_0 \\ 0 \end{bmatrix}. \quad (3)$$

The variables λ_1' and λ_2' are the eigenvalues, $\mathbf{v}_1'$ and $\mathbf{v}_2'$ are the eigenvectors, and c_1' and c_2' are the constants of the simultaneous differential equations in Supplementary Fig. 2a. A detailed definition of these values are provided in the two-compartment solution in the **Detailed computation** section, and are mathematical expressions based on C_0, F, and k_5 and k_6, the latter of which are rate constants governing association and dissociation with the nonspecific compartment.

A similar procedure can be carried out for the three-compartment model associated with the targeted NP:

$$\begin{bmatrix} C_f(t) \\ C_b(t) \\ C_{ns}(t) \end{bmatrix} = c_1[\mathbf{v}_1]e^{\lambda_1 t} + c_2[\mathbf{v}_2]e^{\lambda_2 t} + c_3[\mathbf{v}_3]e^{\lambda_3 t},$$
$$\text{with} \begin{bmatrix} c_1' & c_2' & c_3' \end{bmatrix} = \begin{bmatrix} \mathbf{v}_1' & \mathbf{v}_2' & \mathbf{v}_3' \end{bmatrix}^{-1} \cdot \begin{bmatrix} C_0 \\ 0 \\ 0 \end{bmatrix}; \quad (4)$$

where λ_1, λ_2, and λ_3 are the eigenvalues, $\mathbf{v}_1$', $\mathbf{v}_2$', and $\mathbf{v}_3$' are the eigenvectors, and c_1', c_2', and c_3' are the constants of the simultaneous differential equations in Supplementary Fig. 2b. A detailed definition of these values is provided in the three-compartment solution in the **Detailed computation** section, and are mathematical expressions based on C_0, F, k_3, k_4, k_5, and k_6.

***Ratiometric estimate of binding potential (BP_{Ratio})*:** Now, in the case of the bound compartment for the targeted NP:

$$\frac{dC_b}{dt} = k_3 C_f(t) - k_4 C_b(t). \quad (5)$$

According to the adiabatic approximation (assuming $C_b(t)/C_f(t)$ is constant for all t)[4], then the value of this constant can be evaluated at the boundary condition where $C_b(t)$ attains its maximum ($dC_b(t)/dt = 0$). In general, for the adiabatic approximation to be valid, a suitable time delay between rinsing and imaging should be employed to allow the bound and free concentrations of the targeted NPs to reach equilibrium. If a suitable time delay is not employed, receptor concentration estimation with $BP_{DPM\text{-}NS}$ may be preferable, as it does not require the adiabatic approximation to be valid. However, if valid,

$$\frac{C_b(t)}{C_f(t)} = \frac{k_3}{k_4} \equiv \mathrm{BP} \tag{6}$$

must hold true for all points. Here BP is defined as the "binding potential" and is directly proportional to receptor concentration and the targeted NP's affinity for binding[1]. If BP_{Ratio} is considered [BP_{Ratio} = Targeted NP concentration at end of rinsing/Untargeted NP at end of rinsing – 1[5]], then assuming that the free concentrations of both the targeted and untargeted NPs are the same:

$$\begin{aligned} \mathrm{BP}_{\mathrm{Ratio}} &= \frac{C_{t2}(end) - C_{t1}(end)}{C_{t1}(end)} = \frac{C_f(end) + C_b(end) - C_{f'}(end)}{C_{f'}(end)} \cong \frac{C_b(end)}{C_f(end)} = \mathrm{BP}, \\ &\text{if } C_f(end) \cong C_{f'}(end), \end{aligned} \tag{7a}$$

where "*end*" represents the measurement taken after all tissue rinsing steps, and C_{t1} and C_{t2} are the measured concentrations of untargeted and targeted NPs in the absence of nonspecific binding (**Suppl Fig 2**), respectively. However, with significant nonspecific NP binding, Eq. 7a expands to:

$$\begin{aligned} \mathrm{BP}_{\mathrm{Ratio}} &= \frac{C_{t4}(end) - C_{t3}(end)}{C_{t3}(end)} = \frac{C_f(end) + C_b(end) + C_{ns}(end) - C_{f'}(end) - C_{ns'}(end)}{C_{f'}(end) + C_{ns'}(end)} \\ &\cong \frac{C_b(end)}{C_f(end) + C_{ns}(end)} = \mathrm{BP}\left(\frac{1}{1 + \dfrac{C_{ns}(end)}{C_f(end)}}\right), \\ &\text{if } C_f(end) \cong C_{f'}(end) \text{ and } C_{ns}(end) = C_{ns'}(end), \end{aligned} \tag{7b}$$

where C_{t3} and C_{t4} are the measured concentrations of untargeted and targeted NPs in the presence of nonspecific binding (**Supplemental Fig. 2**), respectively. Therefore, in the presence of a high level of nonspecific binding, this model will underestimate BP owing to a larger denominator value.

Detailed computation

One-compartmental model solution. For the one-compartment model presented in **Supplementary Fig. 1a**, the net flux may be written as:

$$\frac{dC_{f'}}{dt} = -FC_{f'}$$

$$\int_{C_0}^{C_{f'}} \frac{dC_{f'}}{C_{f'}} = \int_0^t F\,dt$$

$$\ln\left(C_{f'}\right) - \ln\left(C_0\right) = -Ft$$

$$C_{t1} = C_{f'} = C_0 e^{-Ft} \tag{8}$$

with C_{t1} representing the total concentration of NP considering all the compartments in the model, and $C_{f'}(t)$ representing the concentration of untargeted NP at time t at initial concentration, C_0.

***Two-compartmental model solution*. Suppl. Figs. 2b** and **3a** represent two scenarios that require two-compartment model solutions, one to denote the free state of the NP while the other denotes the bound state (specific or nonspecific) of the NP. In a general case, let us consider the kinetic parameters to be k_i and k_j. With reference to **Suppl. Fig. 2b**, k_i, k_j will be k_3 ,k_4 while with reference to **Suppl. Fig. 3a** they will be k_5 , k_6. The net exchange rates for the two compartments are as follows:

$$\frac{dC_f}{dt} = -\left(F + k_i\right)C_f + k_j C_k$$
$$\frac{dC_k}{dt} = k_i C_f - k_j C_k \tag{9}$$

Here, $C_f(t)$and $C_k(t)$ denote the concentration of the NP in the free and bound compartments respectively as a function of time t. For simplicity they have been denoted as C_f and C_k. The subscript $k = b$ referring **Suppl. Fig. 2b** and $k = ns'$ referring to **Suppl. Fig. 3a**. These two differential equations can be solved using matrix calculation by representation in the form: where $\mathbf{X}' = \mathbf{AX}$, $\mathbf{X}' = \frac{d}{dt}\mathbf{X}$, $\mathbf{X} = \begin{bmatrix} C_f \\ C_k \end{bmatrix}$,

$\mathbf{A} = \begin{bmatrix} -\left(F + k_i\right) & k_j \\ k_i & -k_j \end{bmatrix}$. The eigenvalues of **A** may be obtained from the relation $\mathbf{A} - \lambda\mathbf{I} = 0$:

$$\lambda_1 = -d \text{ and } \lambda_2 = -e, \tag{10}$$

where, $d = \frac{a-c}{2}$; $e = \frac{a+c}{2}$; $c = \sqrt{a^2 - b}$; $b = 4Fk_j$; $a = F + k_i + k_j$, with corresponding eigenvectors:

$$\mathbf{v_1} = -\frac{1}{\sqrt{\left(k_j/f\right)^2 + 1}}\begin{bmatrix} k_j/f \\ 1 \end{bmatrix}, \mathbf{v_2} = \frac{1}{\sqrt{\left(k_j/g\right)^2 + 1}}\begin{bmatrix} k_j/g \\ 1 \end{bmatrix}, \tag{11}$$

where, $f = F + k_i + d$ and $g = F + k_i + e$. Therefore:

$$\begin{bmatrix} C_f \\ C_k \end{bmatrix} = c_1\mathbf{v}_1 e^{\lambda_1 t} + c_2\mathbf{v}_2 e^{\lambda_2 t},$$
$$\text{with} \begin{bmatrix} c_1 & c_2 \end{bmatrix} = \begin{bmatrix} \mathbf{v_1} & \mathbf{v_2} \end{bmatrix}^{-1}\begin{bmatrix} C_0 \\ 0 \end{bmatrix}. \tag{12}$$

The solution for the signal strength for the targeted NP in the first kinetic model is $C_{t2} = C_f$ and C_b, and for the untargeted NP in the second kinetic model is $C_{t3} = C_{f'}$ and C_{ns}.

Three-Compartmental Model. The three differential equations (**Suppl. Fig 3b**) representing the flux transfer between the three compartments of the previous model for the targeted NP can be solved using matrix methods. These equations may be represented in the form: $X^{'}=AX$ where : $X^{'}=\frac{d}{dt}X$.

Thus, in here, X= $\begin{bmatrix} C_f \\ C_b \\ C_{ns} \end{bmatrix}$ for the targeted NP. Different approaches may be used to solve $X' = AX$ but the one used here is the eigenvalue eigenvector approach. Here, A = $\begin{bmatrix} -(F+k_3+k_5) & k_4 & k_6 \\ k_3 & -k_4 & 0 \\ k_5 & 0 & -k_6 \end{bmatrix}$. Thus on solving the equation A-λI=0 where I is the identity matrix the eigenvalues ($\lambda_1, \lambda_2, \lambda_3$) are obtained and the eigenvectors ($\mathbf{v_1}, \mathbf{v_2}, \mathbf{v_3}$) are obtained using these eigenvalues. Thus the solutions are as follows:

$$\begin{bmatrix} C_f(t) \\ C_b(t) \\ C_b(t) \end{bmatrix} = c_1[\mathbf{v_1}]e^{\lambda_1 t}+c_2[\mathbf{v_2}]e^{\lambda_2 t}+c_3[\mathbf{v_3}]e^{\lambda_3 t}$$

where c_1, c_2, c_3 are obtained from the relation: $[c_1 \quad c_2 \quad c_3] = [\mathbf{v_1} \quad \mathbf{v_2} \quad \mathbf{v_3}]^{-1} \begin{bmatrix} C_0 \\ 0 \\ 0 \end{bmatrix}$. But during inversion, the noise is amplified, so the Kaczmarz method with Tikhonov regularization was applied. Thus,

$$C_{t4}(t)= C_f(t)+C_b(t)+C_{ns}(t)$$

with, $a=-F-k_5-k_3$ $\quad b=-(a-k_4-k_6)$ $\quad r=-(k_4a+k_6a-k_4k_6+k_3k_4+k_5k_6)$

$$d=-(k_4k_6a+k_3k_4k_6+k_4k_5k_6)$$

$$p=r-\frac{b^2}{3} \qquad q=\frac{2b^3}{27}-\frac{br}{3}+d \qquad w=-\frac{1}{2}+\frac{\sqrt{3}}{2}i$$

$$g=-\frac{q}{2}+\sqrt{\left(\frac{q}{2}\right)^2+\left(\frac{p}{3}\right)^3} \qquad h=-\frac{q}{2}-\sqrt{\left(\frac{q}{2}\right)^2+\left(\frac{p}{3}\right)^3}$$

$$y_1=\sqrt[3]{g}\,w+\sqrt[3]{h}\,w^2 \qquad y_2=\sqrt[3]{g}+\sqrt[3]{h} \qquad y_3=\sqrt[3]{g}\,w^2+\sqrt[3]{h}\,w$$

$$\lambda_1=y_1-\frac{b}{3} \qquad \lambda_2=y_2-\frac{b}{3} \qquad \lambda_3=y_3-\frac{b}{3}$$

$$M_1=\sqrt{\left(\frac{k_6+\lambda_1}{k_5}\right)^2+1+\left(\frac{(a-\lambda_1)(k_6+\lambda_1)+k_5k_6}{-k_5k_4}\right)^2}\quad M_2=\sqrt{\left(\frac{k_6+\lambda_2}{k_5}\right)^2+1+\left(\frac{(a-\lambda_2)(k_6+\lambda_2)+k_5k_6}{-k_5k_4}\right)^2}$$

$$M_3=\sqrt{\left(\frac{k_6+\lambda_3}{k_5}\right)^2+1+\left(\frac{(a-\lambda_3)(k_6+\lambda_3)+k_5k_6}{-k_5k_4}\right)^2}$$

$$v_{1a}=\frac{k_6+\lambda_1}{k_5M_1}\qquad v_{1b}=\frac{(a-\lambda_1)(k_6+\lambda_1)+k_5k_6}{-k_5k_4M_1}\qquad v_{1c}=\frac{1}{M_1}$$

$$v_{2a}=\frac{k_6+\lambda_2}{k_5M_2}\qquad v_{2b}=\frac{(a-\lambda_2)(k_6+\lambda_2)+k_5k_6}{-k_5k_4M_2}\qquad v_{2c}=\frac{1}{M_2}$$

$$v_{3a}=\frac{k_6+\lambda_3}{k_5M_3}\qquad v_{3b}=\frac{(a-\lambda_3)(k_6+\lambda_3)+k_5k_6}{-k_5k_4M_3}\qquad v_{3c}=\frac{1}{M_3}$$

$$\mathbf{v_1}=\begin{bmatrix}v_{1a}\\v_{1b}\\v_{1c}\end{bmatrix}\qquad \mathbf{v_2}=\begin{bmatrix}v_{2a}\\v_{2b}\\v_{2c}\end{bmatrix}\qquad \mathbf{v_3}=\begin{bmatrix}v_{3a}\\v_{3b}\\v_{3c}\end{bmatrix}$$

$$s=v_{1a}(v_{2b}v_{3c}-v_{2c}v_{3b})-v_{2a}(v_{1b}v_{3c}-v_{1c}v_{3b})+v_{3a}(v_{1b}v_{2c}-v_{1c}v_{2b})$$

$$V_1=v_{2b}v_{3c}-v_{2c}v_{3b}\qquad V_2=-(v_{1b}v_{3c}-v_{1c}v_{3b})\qquad V_3=v_{1b}v_{2c}-v_{1c}v_{2b}$$

$$c_1=\frac{C_0V_1}{s}\qquad c_2=\frac{C_0V_2}{s}\qquad c_3=\frac{C_0V_3}{s}$$

References

1 Innis, R. B. *et al.* Consensus nomenclature for in vivo imaging of reversibly binding radioligands. *Journal of cerebral blood flow and metabolism : official journal of the International Society of Cerebral Blood Flow and Metabolism* **27**, 1533-1539, doi:10.1038/sj.jcbfm.9600493 (2007).

2 Tichauer, K. M. *et al.* Improved tumor contrast achieved by single time point dual-reporter fluorescence imaging. *Journal of biomedical optics* **17**, 066001, doi:10.1117/1.JBO.17.6.066001 (2012).

3 van den Hoff, J. in *Small Animal Imaging* (eds F. Kiessling & B. J. Pichler) 387-403 (Springer-Verlag, 2011).

4 Lammertsma, A. A. & Hume, S. P. Simplified reference tissue model for PET receptor studies. *NeuroImage* **4**, 153-158, doi:10.1006/nimg.1996.0066 (1996).

5 Liu, J. T. *et al.* Quantifying cell-surface biomarker expression in thick tissues with ratiometric three-dimensional microscopy. *Biophysical journal* **96**, 2405-2414, doi:10.1016/j.bpj.2008.12.3908 (2009).

6 Wang Y., K. A., Som M., Wang D., Chen Y., Leigh S. Y., Meza D., Patrick Z. McVeigh, Brian C. W. , Liu J.T.C. Rapid ratiometric biomarker detection with topically applied SERS nanoparticles. *Technology* **2** (2014).

7 Zavaleta, C. L. *et al.* Multiplexed imaging of surface enhanced Raman scattering nanotags in living mice using noninvasive Raman spectroscopy. *Proceedings of the National Academy of Sciences of the United States of America* **106**, 13511-13516, doi:10.1073/pnas.0813327106 (2009).

8 al, A. A. I. e. Kaczmarz Algorithm For Tikhonov Regularization Problem. *Applied Mathematics.* **13**, 270-276 (2013).

9 Wang, Y. W. *et al.* Rapid ratiometric biomarker detection with topically applied SERS nanoparticles. *Technology* **2**, 118-132, doi:10.1142/S2339547814500125 (2014).

10 Hamzei, N. *et al.* Comparison of kinetic models for dual-tracer receptor concentration imaging in tumors. *Austin J Biomed Eng* **1**, 9 (2014).

11 Rusch, V. *et al.* Overexpression of the epidermal growth factor receptor and its ligand transforming growth factor alpha is frequent in resectable non-small cell lung cancer but does not predict tumor progression. *Clinical cancer research : an official journal of the American Association for Cancer Research* **3**, 515-522 (1997).
